# Existential anxieties and right-wing populism in Europe—why people unconcerned by globalisation vote against it

**DOI:** 10.1007/s12286-023-00569-5

**Published:** 2023-06-12

**Authors:** Anne Metten, Michael Bayerlein

**Affiliations:** 1 Department of Political Science, Kiel University, Westring 400, 24118 Kiel, Germany; 2 Stiftung Wissenschaft und Politik, EU/Europe Research Division, Berlin, Germany

**Keywords:** Populism, Political Parties, Ontological Insecurity, Globalisation, Voting Behaviour, Populismus, Politische Parteien, Ontologische Unsicherheit, Globalisierung, Wahlverhalten

## Abstract

In recent years several contributions have made the argument that right-wing populist voting is interrelated with individuals being negatively affected by globalisation. While there is certainly merit to this argument, it cannot explain why voters unconcerned by globalisation vote for right-wing populist parties. In this article we answer this question and argue that ontological insecurity or, the way we frame it, existential anxieties are a previously overlooked determinant of right-wing populist voting, as these anxieties make people vulnerable to right-wing populist crises narratives even when they are not affected by the crises. Using European Social Survey data for 12 Western European countries between 2004 and 2018, we construct a novel index that measures existential anxieties on the individual level. Our index shows (1) that existential anxieties increase the likelihood of right-wing populist voting and (2) that the fear fuelling narratives of these parties especially mobilise individuals with moderate globalisation attitudes.

## Abstract (de)

In den letzten Jahren wurde in mehreren Beiträgen das Argument vorgebracht, dass die Wahl rechtspopulistischer Parteien damit zusammenhängt, dass Einzelne von der Globalisierung negativ betroffen sind. Auch wenn dieses Argument sicherlich seine Berechtigung hat, kann es nicht erklären, warum Wähler*innen, die von der Globalisierung nicht betroffen sind, für rechtspopulistische Parteien stimmen. In diesem Artikel beantworten wir diese Frage und argumentieren, dass ontologische Unsicherheit oder, wie wir es formulieren, existentielle Ängste eine bisher übersehene Determinante für die Stimmabgabe für rechtspopulistische Parteien sind, da diese Ängste die Menschen anfällig für rechtspopulistische Krisennarrative machen, selbst wenn sie nicht von den Krisen betroffen sind. Unter Verwendung von Daten der European Social Survey für 12 westeuropäische Länder zwischen 2004 und 2018 konstruieren wir einen neuartigen Index, der Existenzängste auf individueller Ebene misst. Unser Index zeigt, dass (1) Existenzängste die Wahrscheinlichkeit erhöhen, rechtspopulistische Parteien zu wählen, und (2) dass die angstschürenden Narrative dieser Parteien insbesondere Personen mit moderaten Globalisierungseinstellungen mobilisieren.

## Introduction

The rise of anti-globalisation right-wing populist parties (RWPP) in Europe has been the focus of a magnitude of contributions in recent years. These contributions often attribute the success of RWPPs to the increased stages of globalisation and negative attitudes towards globalisation (see e.g. Autor et al. 2020; Inglehart and Norris 2016; Rodrik 2018).

Surprisingly, publications have shown that RWPP voting is not necessarily correlated with the degree of economic and cultural globalisation on the national (Bergh and Kärnä 2020) or the individual level (Hays et al. 2019), nor make individual predispositions people more or less adaptive to progressing globalisation (Hainmueller et al. 2015; Margalit 2019; Mutz 2018; Rooduijn 2018). This is supported by the fact that only a minority of RWPP voters hold strongly negative globalisation attitudes.^1^ This means that the success of anti-globalisation RWPPs has in part to be rooted in other factors than negative globalisation attitudes of voters. We address this puzzle and ask why voters that are largely unconcerned by globalisation vote for anti-globalisation RWPPs?

In our answer to this question, we move beyond previous approaches and connect globalisation and populism research to the concept of ontological insecurity. This concept defines the condition of being deeply threatened by societal transition processes that pressure an individual’s identity and sense of security as well as societal stability in connection with the perceived inability of the state to shelter an individual from these transformation processes (see Giddens 1991; Huysmans 1998; Kinnvall 2004). We argue that these ontological insecurities or, the way we frame it, existential anxieties make voters susceptible to crises narratives and securitisation agendas of RWPPs, particularly with regard to progressing globalisation, without holding negative globalisation attitudes nor having a socioeconomic profile of vulnerability. Thus, our analysis draws a clear distinction between anti-globalisation attitudes, which refer to negative beliefs or emotions towards globalisation such as rejection of European integration or immigration, and globalisation losers who have suffered negative economic consequences from globalisation, such as low income, unemployment, or displacement. While anti-globalisation attitudes may be more common among globalisation losers, they are not limited to this group. And this is where our argument comes in, that existential anxieties play a role in the formation of such attitudes, especially if one is objectively not negatively affected by globalisation, i.e., is ‘unconcerned by globalisation’, like the losers just mentioned. To test our arguments, we construct a novel index on existential anxiety using data from the European Social Survey (ESS) in 12 Western European countries between 2004 and 2018.

## Literature review

In this paper we argue that ontological insecurity is a previously overlooked determinant of RWPP voting that works independently of globalisation affectedness. To make this argument, we build on the literature on anti-globalisation attitudes as a determinant of populist voting and the theory of ontological security.

The literature concerned with globalisation attitudes argues that individual predispositions that separate people into ‘winners’ and ‘losers’ of globalisation are a predictor of negative globalisation attitudes (Ardanaz et al. 2013; Kriesi et al. 2006) and right-wing populist voting (Autor et al. 2020; Rodrik 2018; Dippel et al. 2015). According to Kriesi et al. (2006) the ‘losers’ of globalisation are people with low-skills employed in industries that are threatened by offshoring or that face competition in the low-wage and import sector due to increased stages of globalisation (Rommel and Walter 2018; Schaffer and Spilker 2016; Walter 2017). According to the literature, the resulting economic insecurity makes these ‘losers’ of globalisation vote for parties that take policy positions against progressing globalisation (Colantone and Stanig 2018; Hobolt and Tilley 2016). Similarly, economic shocks and decline can cause individuals to adopt authoritarian values and vote for right-wing parties (Ballard-Rosa et al. 2021; Milner 2021).

In contrast to previous findings on the direct effect of economic globalisation on right-wing populist voting, recent research contributions have provided evidence that voters of RWPPs are not fundamentally different in their socio-economic profiles (see, e.g., Lengfeld 2017). According to this literature, RWPPs are predominantly marked by nationalist, xenophobic and populist attitudes (Arzheimer and Berning 2019; Pesthy et al. 2021) in connection with political dissatisfaction and distrust (Rösel and Samartzidis 2018; Schulte-Cloos and Leininger 2022). Bringing globalisation back into the picture, contributions argue that negative globalisation attitudes and RWPP voting are not rooted in the direct effect of economic globalisation but primarily entrenched in the adverse perception of the cultural components of globalisation, i.e. internationalism and multiculturalism (see e.g., Hainmueller et al. 2015). According to this view, RWPP voting is based on the opposition to rising immigrant shares (Becker and Fetzer 2016; Halikiopoulou and Vlandas 2020), increased influx of asylum seekers (Dinas et al. 2019; Dustmann et al. 2019), and perceived loss of national sovereignty (Salgado and Stavrakakis 2019).

Closely connected to the cultural effects of globalisation, the feared loss of identity and cultural hegemony as well as status anxieties are also discussed as determinants of RWPP voting (Gidron and Hall 2017; Margalit 2019). As possible determinants, fear and anxieties open up a new channel on how RWPP voting could be linked to globalisation. Other than the previously discussed direct effects of economic and cultural globalisation, e.g., job losses, wage-cuts, increasing shares of migrants and asylum seekers, fears and anxieties are directed towards the future and not necessarily rooted in objectively discernible features or individual life circumstances. Rather, research contributions suggest a general connection between individual fears as well as anxiety-fuelled campaigns and RWPP voting (Couttenier et al. 2019; Nai 2021).

Hence, according to the literature, people fearing possible negative effects on their life through economic and especially cultural globalisation demand for protection from globalisation processes by a strong Hobbesian state (see Bargetz 2021) and feel that established political parties are unable to control globalisation, making them vote for RWPPs, as these parties promise to put a stop to globalisation and the perceived disruptive transformations associated with it (see Mudde 2013). This disruption of established social orders is at the heart of the literature concerned with ontological security theory. In his seminal contribution Giddens (1984, p. 375) describes ontological security as a “security of being”, i.e. “confidence or trust that the natural and social worlds are as they appear to be, including the basic existential parameters of self and social identity.” While this approach has first been used in sociology and social psychology, recent contributions have transferred the concept to the study of international relations and populism.

Kinnvall (2004, p. 742; see also Kinnvall and Mitzen 2017, p. 7) for example argues that globalisation triggers ontological insecurity, as individuals or collectives of individuals have lost their “stabilising anchor” and their ability to develop a concise and linear narrative of who they are. And, of course, we are experiencing a world that is changing, where there are fewer continuities. One can attribute these changes for the sake of simplicity to globalisation, since it is bringing about processes that are changing the way we live, work and how politics is made, to name just a few examples. These changes can result in feelings of uncertainty, and sometimes perhaps a sense of powerlessness, in the face of crises, wars, and other phenomena that originate in one place but have effects that span the world. Because of the relationship between anti-globalisation attitudes and RWPP voting, recent contributions have connected ontological security via anti-globalisation attitudes to the electoral success of RWPPs (Kinnvall 2018, p. 533; Steele and Homolar 2019, p. 215 ff..). This connection is only logical because RWPPs promise, among other things, to reverse globalisation, to give the state back its former power and to protect the people from external influences. Therefore, we find some recent approaches that combine the theory of ontological security with populism (see e.g. Browning 2019; Steele and Homolar 2019).^2^

However, these contributions are only conceptual in nature or undertake single case studies. That is, a systematic empirical test of whether ontological insecurity actually plays a role in populist attitudes has not yet taken place. Similarly, specific anxieties, in this case ontological insecurity, are not explicitly considered as a possible explanatory factor for a right-wing populist shift in elections alongside status anxiety, social deprivation, and cultural insecurity. Plus, the study of fear as a driver of politically relevant phenomena is extremely intriguing and novel in the field of political science. We now know, for example, that fear has an impact on political participation and voting behavior by influencing attitudes toward crime (Petersen 2010) and terrorism control (Hetherington and Suhay 2011), authoritarianism (Vasilopoulos et al. 2018), party identification (MacKuen et al. 2010), or trust in government in times of the Covid-19 pandemic (Erhardt et al. 2021; see also Bayerlein and Metten 2022).

Thus, our paper builds on the ontological insecurity and the populism literature and identifies an additional component of insecurity and addresses the research gaps in three key aspects. First, the systematic embedment of the concept of ontological insecurity in the causal mechanism of RWPP voting and anti-globalisation attitudes. Second, the utilisation of survey data to construct an index to empirically capture ontological insecurity on the individual level. Third, the use of econometric models to analyse the connection between ontological insecurity and RWPP voting. Before constructing the index, we first explain how ontological insecurity is linked to RWPP voting. In the course of the paper, we incidentally draw a conceptual distinction from ‘ontological insecurity’, the original concept that is used in political science particularly in the field of international relations. For our reception we use the term ‘existential anxiety’, which was also used by Giddens (1991, p. 39), as it is more commonly understandable and clarifies that we refer to the original concept, but adapt it. In particular, in that we will measure it quantitatively.

## The concept of existential anxiety

Our concept of existential anxiety is based on the theory of ontological security. In its original version put forward by psychiatrist Ronald David Laing (1960), ontological insecurity refers to a pathological condition of not being able to keep up with societies’ expectations. Focusing on this deficit, Laing explored the interplay between society and mental health, emphasising the importance of conceiving of oneself in this world as “real, alive, whole” and “a continuous person” (ibid., p. 39).

Giddens (1984, 1991) transferred Laing’s concept of ontological security to sociology and social psychology to describe the trust that most people have in the continuity of their identity and the constancy of the social and material environment surrounding them. Giddens (1991) describes this trust as an “*emotional inoculation* against existential anxieties” (ibid., p. 39) that, when destabilised, leads to ontological insecurity. He attributes this destabilising process in particular to modernity, which tends to eliminate the sense of security usually provided by the community and traditional settings. Hence, ontologically insecure people identify modernity as a root cause of their anxieties and attempt to ‘de-modernise’ to reverse the “trends that have left the individual ‘alienated’ and beset with the threats of meaninglessness” (Berger in Pathak 1998, p. 22).

Later, Huysmans (1998) as well as Kinnvall (2004) introduced the concept of ontological insecurity to international relations research by emphasising a direct connection to state and politics. This connection addresses one of the premises of international relations: the legitimacy and function of states. This legitimacy and function rests on a state’s ability to provide security and order and is accompanied by internal homogenisation and the delimitation of national identity externally. The ‘other’ can become a threat in this context, since it can call into question the order within a state (Huysmans 1998, p. 242; Kinnvall 2018, p. 537).

The perceived disruption of the traditional order, the impression of chaos, and the threat of the ‘other’ links the concept of ontological insecurity to globalisation processes, as globalisation “challenges simple definitions of who we are and where we come from” (Kinnvall 2004, p. 742). What is at issue here is the subjective experience of anxiety of change—be it objectively rational or irrational (Mitzen 2006, p. 346). In that, this paper does not attempt to explore how these fears emerge individually. We already know that individual psychological factors can be attitude amplifiers, allowing globalisation to trigger rising authoritarianism, declining trust, insecurity, and status anxiety among individuals. For instance, Stephan and Stephan (2000) have shown how perceived threats influence prejudice between social groups, Duckitt (e.g. Sibley and Duckitt 2013) in his extensive work on the relationship between prejudice and ideology and the adoption of authoritarian values and Ballard-Rosa et al. (2021) or Hetherington and Suhay (2011) on how these translate into populist voting.^3^ The concept of status anxiety is also an integral part of populism research. In particular, Gidron and Hall (2017) have shown how influential it is on the electoral choice of populist parties. However, status anxiety, unlike ontological insecurity, is an individual phenomenon that is not related to societal changes in general, but to individual perceptions of one’s hierarchical position in the socioeconomic structure (see ibid. and Chap. 3.2 for further details on the difference). In this paper, however, the focus lies on analysing the impact of the socio-psychological phenomenon of ontological insecurity, i.e., the interplay between individual anxieties activated by societal circumstances, on the rise of anti-globalisation RWPPs. This is because, unlike, and in addition to the differences mentioned above, authoritarian attitudes or status anxiety, for example, ontological insecurity involves an inside and an outside view. That is, it involves psychological factors that interact with social phenomena.

For, taking into account the early and later receptions of ontological security, two analytical dimensions emerge that constitute this interaction. First, an *intersubjective *or* exogenous *dimension, where individuals “are concerned with maintaining a consistent notion of self to enhance their ontological security with other[s]” (Kinnvall et al. 2018, p. 252; see also Giddens 1991, p. 33). This involves maintaining self-concepts and identity security, especially in interaction with other people, which is achieved through routines and stability (Kinnvall and Mitzen 2017, p. 6). This dimension in particular expresses the fear of social change. The biggest social changes over the last decades are associated with globalisation (cf. Ballard-Rosa et al. 2021, p. 4). As mentioned before, and as is typical with fears, it is not important here whether one is actually affected by “fundamental shifts in production capacity and employment patterns; economic changes from trade shocks […] [or] technological change […]” (ibid.). What matters is how one perceives this threat scenario and whether there are external influences that reinforce these fears—as we will see in the next chapter.

Second, an *intra-subjective* or *endogenous *dimension, in which the state serves “as a provider of ontological security for its citizens” (Kinnvall et al. 2018, p. 252; see also Kinnvall and Mitzen 2017, p. 6). Conversely, individuals feel more existential anxiety when they perceive the state as unwilling or unable to provide security. So, the question here is who will ensure that protection is provided. States offer in a special way and qua their elementary existence a protective mantle that is supposed to guarantee security. Crisis situations, which happen in individual states and/or have worldwide effects, challenge this protective mantle or damage it. Routines are thus forced to adapt, which can trigger anxiety that can lead to regressive behavior, followed by attempts to restore routines and regain cognitive control over the changed environment (cf. Ejdus 2018, p. 887). Here, anxiety can no longer be controlled and “ontological security comes under immediate strain” (Rumelili 2016, p. 11). This endogenous dimension of ontological security as well as the exogenous dimension are both strongly linked to the essence that defines RWPPs.

### Linking existential anxieties to right-wing populist voting

The two components that link RWPP voting to existential anxieties are rooted in the populist rhetoric itself as well as the right-wing populist host ideology employed by RWPPs. In defining populist parties we first follow Cas Mudde (2004, p. 543) and argue that populism as a thin ideology considers society to be “separated into two homogeneous and antagonistic groups, ‘the pure people’ versus ‘the corrupt elite’, and which argues that politics should be an expression of the volonté générale (general will) of the people.” On the one hand, the thin-ideology of populism itself already appeals to people that experience existential anxieties. Previous contributions have shown that voters are attracted to populist parties based on their (perceived) lack of representation and the failure of political elites to provide policy solutions to pressing problems (Castanho Silva and Wratil 2023; Hawkins et al. 2020; Huber et al. 2023). Since existential anxieties are linked to the feeling not being protected against identity-threatening progressing globalisation, we identify a direct connection to the determinants of populist voting identified by previous contributions.

On the other hand, the host ideology of populist parties carries substantial importance since it is the host that determines what exactly the ‘will of the people’ is. The host ideology of RWPPs is a combination of national self-interest and identity, traditional culture, authority, and anti-cosmopolitan views (see Inglehart and Norris 2016; Mudde 2013). In combining these issues, most RWPPs have moved beyond the single-issues character of old radical right-wing parties and distinctly established themselves as anti-globalisation parties with a comprehensive set of reactionary policy positions directed towards halting societal changes and returning a country as well as its society to their former greatness (cf. Bayerlein 2021; Zaslove 2008). However, anti-globalisation attitudes and even anti-migration attitudes are also sometimes associated with left-wing populist parties (Santana and Rama 2018) although these parties frame their populist discourse—by definition—in economic terms by pitting a capitalist financial elite against an exploited work-force of ordinary people. While non-right-wing populist parties could also benefit from rising existential anxieties, ontological insecurity directly relates to the construction of one’s identity within the nation state, which is predominantly addressed by RWPPs.

In addition to divisive ‘us vs. them’ rhetoric, RWPPs’ coherent anti-globalisation and reactionary policy positions are communicated and strategically implemented by means of crises narratives and securitisation . What is important here is that these need not be objective crises but perceived crises, as “populist actors actively perform and perpetuate a sense of crisis” (Moffitt 2015, p. 195; see also Mitzen 2006, p. 346; Huysmans 1998; Kinnvall 2018; Steele and Homolar 2019). The crisis is being conjured up as a permanent condition giving rise to a direct strategy of populists to securitise. This securitisation happens “when an issue is presented as posing an existential threat to a designated referent object” (Buzan et al. 1998, p. 21). By occupying globalisation issues and defining globalisation in and by itself as existential threats to society, culture, religion, and tradition, populists securitise globalisation processes (cf. Huysmans 1998, p. 242) and stress the need for quick action to halt allegedly dangerous societal changes (Taggart 2004, p. 275). A need for action that is—for selfish reasons—not addressed by the ‘ruling elite’ of the political system, but only by the RWPP (cf. Sahin 2021, p. 6).

Crisis narratives and securitisation “can temporarily strip individuals of their presumed biographical continuity, sparking episodes of anxiety and ontological insecurity” (Homolar and Scholz 2019, p. 356). Anxiety, therefore, is what directly links ontological insecurity to anti-globalisation RWPPs (cf. Steele and Homolar 2019, p. 215), as these parties fuel both dimensions of existential anxiety. First, in the impression or articulation of disorder or chaos both induced by modernity, as well as the attempt to restore order by turning back time. Second, the perception that the state does not prevent this nor established parties are capable or willing to manage the crisis situation. Inglehart and Norris (2016, p. 11) also argue along these lines when summarizing the general sentiment on the issue of the rise of populism: “Anxiety arising from contemporary events—boatloads of migrants and refugees flooding into Europe, images of the aftermath of random acts of domestic terrorism in Paris, Brussels, and Istanbul, and austerity measures—are blamed for exacerbating economic grievances linked with rising income inequality, the loss of manufacturing jobs, and stagnant wages.” In an increasingly crisis-ridden world, the sense of security and continuity can be so challenged that the aforementioned routines turn into the opposite. That is, routinized behaviors and practices that psychologically make it possible to make the otherwise chaotic world predictable and manageable become so entrenched and hardened that they turn into ignorance. “Ignorance [then] becomes a coping mechanism, creating an opening for populist rhetoric that portrays the rejection of facts and knowledge as an assertion of autonomy over elites” (Steele and Homolar 2019, p. 215).

Irrespective of this reasoning, the question is how our existential anxiety approach relates to other approaches that try to explain (right-wing) populist voting, namely attitude-based approaches and fast-emerging body of literature concerned with social alienation and social status anxieties as a determinant of populist voting. Concerning the attitude- or value-based determinants of populist voting, we argue that our approach complements the economic and cultural determinant mechanisms proposed by the literature in that existential anxieties precede the formation of specific attitudes and can but do not have to be correlated with these attitudes. Specifically, we base this argument on recent publications that show how psychological and emotional dispositions like agreeableness and anger relate to populist voting irrespective of specific attitudes that might nonetheless be correlated with these dispositions (Bakker et al. 2021b; Rhodes-Purdy et al. 2021).

### Stylised facts on existential anxieties and hypotheses

Other than in case of the attitudinal approaches to explain RWPP voting, existential anxieties do not precede status anxieties and authoritarian values, but are complementary phenomena. This is the case for three reasons. First, status anxieties are individual psychological preconditions that are related to a declining position in the societal hierarchy felt by the individual (Bolet 2021; Gidron and Hall 2017). In that sense, status anxieties are rooted in the fear of losing the hierarchical position in society once held due to one’s statues, while existential anxieties are centred around maintaining self-concepts and identity security. Second, authoritarianism revolves around three subdimensions, namely authoritarian aggression, submission, and conventionalism (Altemeyer 1981). In other words, authoritarian individuals tend, for example, to use force to enforce their demands, whereas, on the other hand, they are loyal to people with a higher social status and tend to abide by rules (cf. Ballard-Rosa et al. 2021, p. 6). Thus, there are clear differences with our concept of existential anxiety, which are also evident in the subsequent empirical measurement of our concept, although similarities can also be observed. Here, however, we refer to the literature on authoritarianism, which provides a concrete and different empirical measurement (see Ballard-Rosa et al. 2021; Hetherington and Suhay 2011). Third and even more important, status anxieties and authoritarian values are by definition individual phenomena that do not relate to societal changes in general. This is amplified by the finding of Gidron and Hall (2017) who show that individuals with a low social status show a reduced likelihood of right-wing populist voting, as one has to have a moderate social status to be afraid of losing it. However, the probability of voting for left-wing populist parties could be even enhanced by having a low social status. In comparison to status anxiety, our mechanism works on the level of societal changes and the perception of these changes as dangerous irrespective of individual affectedness, status concerns or alienation. From this follows that we expect to see a direct connection between an individual’s existential anxiety and the individual anti-globalisation RWPP voting probability.

#### Hypothesis 1

The higher the level of existential anxieties, the higher the likelihood of RWPP voting.

This hypothesis is in the first instance formulated unidirectionally. Likewise, it may be that political attitudes not only influence RWPP voting, but also that these parties reinforce or cause the salience of existential anxieties. There is evidence that political attitudes and psychological constructs are not causally prior to vote choice or political attitudes. For example, Rooduijn et al. (2016) show that political discontent is both a cause and a consequence of the rise of populist parties, or Harteveld et al. (2017) that voters position themselves along party lines on a given issue. In line with this, Bakker et al. (2021a) have most recently shown that the relationship between personality and policy preferences is not unidirectional, but at least reciprocal. We keep this possibility of reciprocity in mind and will address reverse causality in the robustness checks.

**Fig. 1:**
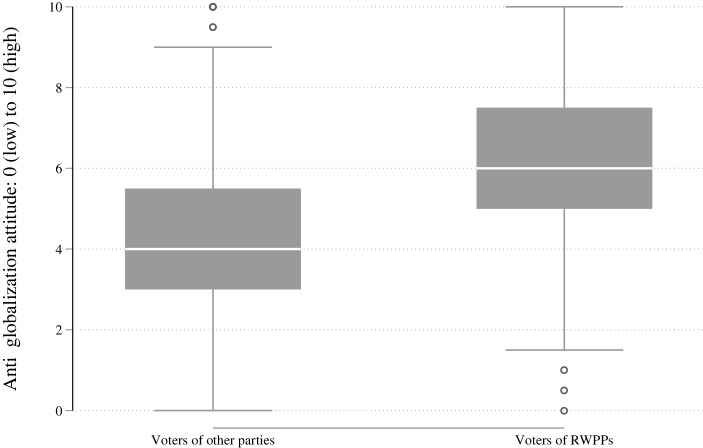
Globalisation attitudes of RWPP and voters of other parties

While the first hypothesis addresses the expected correlation between existential anxieties and RWPP voting, we want to take our proposed theoretical mechanism one step further and focus in detail on the voters without strong anti-globalisation attitudes. As outlined above, negative consequences of globalisation and the backlash against them is correlated with populist voting (Autor et al. 2020; Dippel et al. 2015; Rodrik 2018). Based on this, we reason that people with very negative globalisation attitudes have a high likelihood of RWPP voting with little impact of the individual existential anxieties, and vice versa. In contrast to this and of particular interest here are voters without such negative attitudes, i.e., voters with moderate or even positive globalisation attitudes. To illustrate the globalisation attitudes of voters, we plot these attitudes depending on the vote choice indicated by the respective respondents in Fig. 1.

While the voters of RWPPs have more negative attitudes toward globalisation on average, the overlapping interquartile range in the figure also strikingly underscores that a large share of RWPP voters have moderate or positive attitudes toward globalisation. Rather, the explanatory power of existential anxieties lies in the middle of the distribution. The question, then, is how people with moderate attitudes are influenced by existential anxieties.

To answer this question, we argue that these voters are not per se drawn towards RWPPs based on their anti-globalisation attitude but become susceptible to RWPP crises narratives if they experience existential anxieties irrespective of their globalisation attitudes. It is especially this group of voters, which is largely unconcerned by globalisation, whose voting behaviour is of key interest to this paper, as these are the people that vote for anti-globalisation RWPPs irrespective of the fact that they do not hold negative globalisation attitudes in the first place. We are talking about people who in principle do not oppose international trade, the EU, or foreigners. Rather, they have a diffuse fear that can be addressed by RWPPs. Our argument to explain this voting behaviour is that RWPPs provide a sense of security to this people that is independent of the specific attitudes towards globalisation.

To makes this argument we build on the reasoning and evidence of Hetherington and Suhay (2011) as well as Vasilopoulos et al. (2018). They show that individuals who do not have strong authoritarian dispositions make more restrictive and aggressive policy choices due to perceived threats to their security than those individuals that already have strong authoritarian predispositions. Translated to our case, this means that individuals with strong and predetermined globalisation attitudes are less likely to have their policy choices altered by insecurity scenarios. From this follows that people with negative globalisation attitudes will vote for RWPPs due to the congruence of policy positions and preference irrespective of their individual anxiety levels.

However, people with high levels of anxiety are not per se ideologically close to the anti-globalisation policy position of RWPPs but can be mobilised to vote for these parties if RWPPs are able to fuel their existential anxieties without necessarily changing their globalisation attitudes. In that regard the vote choice becomes not a question of attitude but of anxiety. With this approach, we argue that the translation of policy preferences into vote choices is moderated by the degree of existential anxiety with high levels of anxieties resulting in a higher probability of voting for anti-globalisation RWPPs, especially when globalisation attitudes are not extremely negative. Based on this we hypothesise the following.

#### Hypothesis 2

The correlation of existential anxieties and RWPP voting is largest when the anti-globalisation attitudes are moderate.

## Data and operationalisation

We test our hypotheses by developing the first empirical index that captures existential anxieties. To construct our index, we use survey data from the last eight rounds of the ESS (2019). We chose to use the ESS because it is one of the most comprehensive European surveys that since 2002 repeatedly employs the same questionnaires in over 30 countries. Our proposed index construction is nonetheless open to other surveys.

The constructed index is utilised in 12 Western European countries^4^ between 2004 and 2018 to predict the RWWP voting probability on the individual level. Our sample is limited to 12 Western European states between 2004 and 2018 for three reasons. First, we use the ESS from 2004 onwards since the first round is missing questions. Second, we limited the sample to states in which RWPPs ran for national elections, as non-populist voting is not independent from the existence of RWPPs (see Hernández and Kriesi 2016).^5^ Third, we excluded Eastern European states because they differ strongly in their propensity towards RWPPs (Gunnarsson and Zoega 2017; Minkenberg 2002).^6^

### Constructing the existential anxiety score

For the 12 states we have compiled an index, the *Existential Anxiety Score* (EAS), which measures the existential anxiety of roughly 105,000 respondents on a scale from 0–10, with higher values denoting greater anxiety. The EAS was constructed in three steps.

First, the variable selection for our EAS is based on the ontological insecurity theory’s two core dimensions. For the *exogenous* dimension, which revolves around the need for security and stability, we have included three variables from the ESS. These variables capture the individual need for safe and secure surroundings (*safety*), the extent to which they feel that their state should ensure their safety against threats (*protection*) is needed, and importance they attach to customs and traditions (*traditions*). We argue that the attachment to traditions is a vital component in ontological insecurity, as people that place great importance on societal customs will inevitably see these customs threatened by societal changes. It is important to note that this component of the exogenous dimension can also just capture the appeal of the right-wing host ideology, i.e., conservatism. We control for this, by dropping the traditions component from the EAS index in the robustness checks.

To capture the *endogenous* dimension, we use three variables to measure the perception that the political elite cannot be trusted to fulfil the state’s task of providing security. These variables are trust in parties (*trustparty*), politicians (*trustpol*) and in parliament (*trustparl*). The endogenous dimension also poses the risk of just capturing anti-elite populist attitudes and not the specific interaction between the individual need for security (exogenous dimension) and the trust in the states capability to provide this security (endogenous dimension). We address this methodological issue in the course of the robustness checks by running additional regression models that drop the endogenous dimension from the EAS and that address the individual effects of the two dimensions. What is important here is that we are concerned with measuring the latent construct of existential anxieties. Thus, individual components, such as nationalism or anti-elitism, may well merge into existential anxieties as we operationalize them. Consequently, in order to capture the latent construct of existential anxieties closely along theoretical accounts of ontological insecurity, we cannot avoid harnessing existing features that are a fixture in the literature on RWPPs, but combining them in new ways.

Based on this a total of six variables from the ESS is used to construct our EAS.^7^ Since the scales of the six variables vary, we panel normalised them with the formula:

1 $$Vn=\frac{Vi-Vmin}{Vmax-Vmin}*10,$$

where *Vn* is the normalised value of the respective variable (*V*) for an individual (*i*), and *Vmax* and *Vmin* being the minimum and maximum values of the respective variable. Based on this normalisation, the variables are rescaled to range from 0–10.^8^

The second step involved a statistical evaluation of the relationship among the variables with four subsequent principal component analyses (PCA) because this procedure is a well suited approach in measuring latent constructs (Gwartney and Lawson 2001, p. 7).^9^ The PCA decomposes the variance of variables that are thought to best represent a latent construct, in our case existential anxiety. From this decomposition a new linear weighted combination of variables is created. This weighted combination then accounts for the largest possible amount of variance in the original data (Vyas and Kumaranayake 2006, p. 460). Table 1 shows the results of the first PCA for the six variables.

**Table 1:** Eigenvectors of the principal component analysis

Variable	Component 1	Component 2	Unexplained
Safety	0.0975	*0.5947*	0.4028
Traditions	0.0043	*0.5201*	0.5617
Protection	0.0862	*0.6020*	0.3941
Trustpol	*0.5876*	−0.0785	0.1117
Trustparl	*0.5486*	−0.0426	0.2316
Trustparty	*0.5804*	−0.0733	0.1345
Variation explained	0.4240	0.2700	–

Eigenvectors with values above 0.35 are in italics. These load most strongly on the respective components. Adding up the explained variation, the cumulative variation explained is 0.6939

From the table it becomes apparent that two linear combinations or components explain the largest variance in the data. This can be seen from the fact that the first two components have an eigenvalue above 1—a threshold originating from the Kaiser-Guttman rule, according to which only those components that explain more variance than the original variables, i.e. variables with an eigenvalue above 1 should be retained (Vyas and Kumaranayake 2006, p. 460). Overall, the first two components explain about 70% of the variance in the data. Plus, the three variables that we have assigned to the endogenous dimension based on theoretical considerations, strongly load on the first component. The second component is dominated by the variables assigned to the exogenous dimension. In other words, the respective three variables are interrelated but each group represents somewhat different latent sub-constructs. The theory motivated division into an exogenous and endogenous dimension therefore appears reasonable.

Based on this finding, we do not simply combine the six variables in one PCA but sub-divide our index construction into two PCAs one for each of the two dimensions. Running individual PCAs for the three respective variables of the *exogenous* and the *endogenous* dimension produced the weights displayed in Eqs. 2 and 3. The two dimensions are then combined additively in Eq. 4.^10^ All indicators aggregated are normally distributed, have been standardised, and range from values between 0 and 10, with higher values indicating greater existential anxiety.

2 $$\textit{Exogenous}=0.6058*\textit{safety}+0.5101*\textit{traditions}+0.6105*\textit{protection}$$

3 $$\textit{Endogenous}=0.5933*\textit{trustpol}+0.5521*\textit{trustparl}+0.5858*\textit{trustparty}$$

4 $$EAS=\frac{\textit{exogenous}+\textit{endogenous}}{2}$$

### RWPP voting and globalisation attitude

The RWPP voting variable is a binary variable indicating whether an individual voted for an RWPP in the last national election. We use the ESS item that asks respondents who they voted for in the last national election. The employed identification strategy of RWPPs is based on the coding of far-right populist parties of the *PopuList* by Rooduijn et al. (2019). Due to our specific focus on anti-globalisation parties, we use the Chapel Hill Expert Survey (CHES) to only include RWPPs with an anti-globalisation policy position. The anti-globalisation positions are determined by calculating the country specific distributions of policy positions on the EU integration and immigration dimension and coding the parties in the upper 25th percentile of the distribution as anti-EU or anti-immigration. If both categories are given, we code the party as anti-globalisation. Based on the PopuList in combination with the CHES, we identified 18 anti-globalisation RWPPs in our sample countries (see Table 5 in the Appendix).

To code the individual globalisation attitude, we created a proxy variable from the ESS dataset. This proxy is composed of two ESS questions that tap into different aspects of economic and cultural globalisation. The two questions are whether European unification has gone too far (*eutoofar*) and whether the country’s cultural life undermined by immigrants (*imueclt*). We again used panel normalisation (see Eq. 1) to make the variables run from 0–10. Despite being not included in the ESS, we distinctly chose measures of cultural and political globalisation and not measures of economic globalisation. This is the case because our contribution focuses on existential anxieties and threatened individual identities rooted on the cultural and political realm of the nation state, which is again directly related to the policy positions of RWPPs. While economic dimensions can also be of importance, they are so primarily in the context of analysing left-wing populist voting. The globalisation attitude variable is estimated calculating the average score from both questions.

### Control variables

In addition to the three main variables, we also introduce five categories of control variables (see Table 4 in the Appendix for a detailed description): (1) *demographic variables *(‘dem’) are employed because previous studies have shown that men differ systematically from women (*gender*), older from younger people (*age*), rural from urban citizens (*domicil*), and religious from secular people (*relgious*) in their attitudes and policy preferences (see e.g. Arzheimer and Carter 2009; Iversen and Soskice 2001; Walter 2017). Each of the former is more likely to have a negative attitude towards globalisation and a higher likelihood of voting RWPPs. Similar we introduce (2) *socioeconomic variables* (‘socec’), which account for the objective effects of globalisation on individuals. Based on previous research, we reason that people who have rather low household incomes (*income*), are not union members (*union*), are less educated (*eduyears*), and/or are unemployed (*unemp*), are more likely to adhere to anti-globalisation ideas or parties (see e.g. Gidron and Hall 2017; Rodrik 2018).

The fear of losing one’s job because it could be offshored or import competition can influence policy preferences (Autor et al. 2020; Rommel and Walter 2018; Walter 2017). Therefore, we have coded the (3) *labor market risk variables* (‘labr’) with the help of Walter (2017) indicating whether a person works in a sector that is easily offshorable (*offshore*) and/or an exporting sector (*tradeable*). To account for (4) *status anxiety *(‘status’) and inspired by Paskov et al. (2015), we use a status seeking variable (*statusseek*) to proxy for social status anxieties (see e.g., Wilkinson and Pickett 2010). Second, we control for the household income perception (*incomefeel*) and the attitude towards policies aimed at reducing income differences (*inequality*) (see e.g., Hacker and Pierson 2020; Rodrik 2021). With (5) *political opinion variables* (‘polit’), we control for self-placement on a left-right scale (*lrscale*) and the satisfaction with democracy (*satisdem*) (see e.g., Gunnarsson and Zoega 2017; Rommel and Walter 2018). To control for economic and cultural globalisation (see e.g., Autor et al. 2020; Dippel et al. 2015; Rodrik 2018), we employ country-fixed effects. We hence test whether the effect of existential insecurity is (in‑)dependent of being objectively affected by globalisation.

### Method

The two hypotheses derived above will be tested with two distinct regression equations. To test the first hypothesis, we regress the RWPP vote variable on the EAS. Since the vote variable is coded binary, we use a logistic regression to calculate the probability of RWPP voting dependent on the EAS. Our baseline model is defined by:

5 $${Pr\left(RWPPVote=1| x\right)}_{i}^{c{,}t}=\frac{e^{\alpha +\beta {\mathrm{EAS}}_{i}^{c{,}t}+{\beta _{j}}{X}_{j}^{c{,}t}+{\gamma _{c}}+{\lambda _{t}}+\varepsilon }}{1+e^{\alpha +\beta {\mathrm{EAS}}_{i}^{c{,}t}+{\beta _{j}}{X}_{j}^{c{,}t}+{\gamma _{c}}+{\lambda _{t}}+\varepsilon }},$$

where *RWPP Vote* is the binary variable indicating whether an individual (*i*) in a country (*c*) at a given year (*t*) voted for an RWPP in the last election as a function the existential anxiety score (*EAS*) of the same individual. Further, 𝒳 gives a vector of the described set of control variables (*j*), while the additional terms give the constant (*α*) as well as country (*γ*_*c*_) and year (*λ*_*t*_) fixed effects.

In order to test the second hypothesis, according to which the effect of existential anxieties on the RWPP voting probability is highest when globalisation attitudes are moderate, we model an interaction term between our EAS variable and the globalisation attitude (*Attitude*). We again use a logistic regression. Our baseline model is:

6 $${Pr\left(RWPPVote=1| x\right)}_{i}^{c{,}t}=\frac{e^{{\alpha +\beta _{1}\mathrm{EAS}}_{i}^{c{,}t}+{\beta _{2}\text{Attitude}}_{i}^{c{,}t}+{\beta _{3}(\mathrm{EAS}}_{i}^{c{,}t}*{\text{Attitude}}_{i}^{c{,}t})+{\beta _{j}}{X}_{j}^{c{,}t}+{\gamma _{c}}+{\lambda _{t}}+\varepsilon }}{1+e^{{\alpha +\beta _{1}\mathrm{EAS}}_{i}^{c{,}t}+{\beta _{2}\text{Attitude}}_{i}^{c{,}t}+{\beta _{3}(\mathrm{EAS}}_{i}^{c{,}t}*{\text{Attitude}}_{i}^{c{,}t})+{\beta _{j}}{X}_{j}^{c{,}t}+{\gamma _{c}}+{\lambda _{t}}+\varepsilon }},$$

where everything is identical to the above logit regression (Eq. 5) except for the interaction term between the EAS and the globalisation attitude of the same individual.

## Results

The regression analysis that is employed to test the first hypothesis is displayed in Table 2. Model 1 reports the coefficient of the independent variable with year- and country-fixed effects. The coefficient is with 0.452 positive and statistically significant, indicating a positive correlation between existential anxiety and negative globalisation attitudes.

**Table 2:** Regression results for EAS predicting RWPP voting

Main variables	(1)	(2)	(3)	(4)	(5)	(6)
EAS	0.452*** (0.01)	0.481*** (0.01)	0.449*** (0.01)	0.454*** (0.01)	0.462*** (0.01)	0.336*** (0.01)
*Control variables*						
Dem	–	✓	✓	✓	✓	✓
Socec	–	–	✓	✓	✓	✓
Labr	–	–	–	✓	✓	✓
Status	–	–	–	–	✓	✓
Polit	–	–	–	–	–	✓
Country Fixed Effects	✓	✓	✓	✓	✓	✓
Year Fixed Effects	✓	✓	✓	✓	✓	✓
Observations	105,808	105,410	95,306	85,098	83,287	81,424
Pseudo R^2^	0.124	0.138	0.150	0.151	0.154	0.219

Significance levels: *** *p* < 0.01, ** *p* < 0.05, * *p* < 0.10

Robust standard errors in parentheses

Model 2 introduces the first group of control variables with the ‘dem’ variables. Including these variables slightly increases the coefficient to 0.481. The following models introduce the additional groups of control variables. The introduction of the ‘socec’ control variables reduces the size of the coefficient to 0.449. The addition of ‘labr’ and ‘status’ control variables does not change the coefficient. However, including the ‘polit’ control variables decreases the size of the coefficient to 0.336, showing that people’s left-right placement and satisfaction with democracy carries some predictive power. Across the models the sample size is shrinking with the introduction of every group of control variables due to missing observations. In total, the regression analysis shows that there is a stable and significant and positive correlation between our EAS and the individual anti-globalisation RWPP voting probability.

**Fig. 2:**
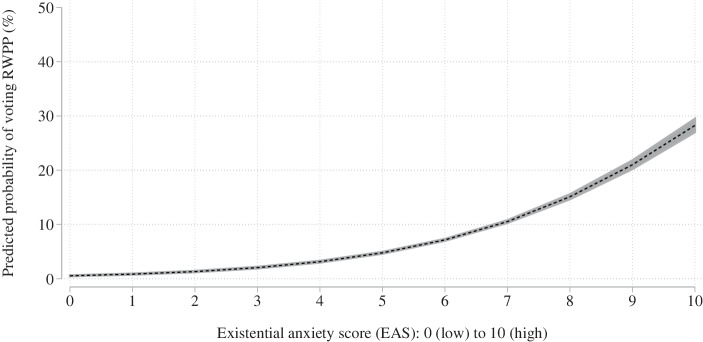
Predicted probabilities of RWPP voting calculated from EAS

To address the substantial relevance of this correlation, we calculate the effect size, ceteris paribus, of a 1 standard deviation increase of the EAS at the mean. To include all observations, the marginal effects are calculated based on Model 1, since this model controls for individual years and countries in the sample and uses the full set of observations.^11^ If the EAS is increased by 1 standard deviation the RWPP voting probability increases by about 0.21 standard deviations. To illustrate this correlation Fig. 2 plots the marginal effects of the regression analysis based on Model 1. The figure displays a strong and positive correlation between existential anxiety and the RWPP voting probability, supporting our first hypothesis in statistical and substantial terms.

In order to test the second hypothesis, we include the interaction term between the EAS and the globalisation attitude variable. The results of the logistic regression are shown in Table 3. The coefficient of the EAS variable in Model 1 is with 0.574 positive and statistically significant again indicating correlation with the RWPP voting probability. As expected, the coefficient of the globalisation attitude variable is also positive and statistically significant with 0.789.

**Table 3:** Regression results for interaction term predicting RWPP voting

Main variables	(1)	(2)	(3)	(4)	(5)	(6)
EAS	0.574*** (0.03)	0.615*** (0.03)	0.590*** (0.03)	0.586*** (0.03)	0.598*** (0.03)	0.540*** (0.04)
Attitude	0.789*** (0.03)	0.788*** (0.03)	0.746*** (0.03)	0.741*** (0.04)	0.740*** (0.04)	0.678*** (0.04)
EAS × Attitude	−0.057*** (0.00)	−0.058*** (0.00)	−0.055*** (0.01)	−0.054*** (0.01)	−0.054*** (0.01)	−0.055*** (0.01)
*Control variables*						
Dem	–	✓	✓	✓	✓	✓
Socec	–	–	✓	✓	✓	✓
Labr	–	–	–	✓	✓	✓
Status	–	–	–	–	✓	✓
Polit	–	–	–	–	–	✓
Country Fixed Effects	✓	✓	✓	✓	✓	✓
Year Fixed Effects	✓	✓	✓	✓	✓	✓
Observations	91,204	90,873	82,569	73,979	72,417	71,032
Pseudo R^2^	0.196	0.205	0.208	0.210	0.211	0.253

Significance levels: *** *p* < 0.01, ** *p* < 0.05, * *p* < 0.10

Robust standard errors in parentheses

More important, the interaction term that addresses the theorised connection between existential anxieties and globalisation attitudes is with −0.057 negative and statistically significant. The negative sign indicates that the interaction effect is not equally large across different combinations of the values of the independent variables. Rather, the effect is smaller at the extremes of the distribution of the globalisation attitude variable than in the middle of the distribution.

The interaction term remains stable and significant with −0.058 when the first set of control variables is included in Model 2. The size of the coefficient slightly drops to −0.055 with the inclusion of additional control variables in Model 3. From this the interaction term’s coefficient does hardly change in size and remains statistically significant across the additional models. In total, this strongly supports our second hypothesis according to which we expect the effect of existential anxieties on RWPP voting to be larger when the globalisation attitudes are moderate.

To interpret the coefficient of the interaction term in substantial terms, we again assess the predicted probability. We calculate the effect size, ceteris paribus, of a 1 standard deviation change in the mean EAS in three different anti-globalisation attitude scenarios (low = 0, moderate = 5, high = 10). We again use Model 1 to include all observations. In the scenario of a low anti-globalisation attitude (Attitude = 0) a 1 standard deviation increase in the EAS leads to a 0.04 standard deviation increase in the RWPP voting probability. In a moderate anti-globalisation attitude scenario (Attitude = 5) the same increase in the EAS results in an about 0.12 standard deviation increase in the RWPP voting probability. This indicates that the effect of the same change in the EAS is almost three times as large in case of a moderate anti-globalisation attitude than in case of a low anti-globalisation attitude. In line with our expectation a 1 EAS standard deviation increase only results in a 0.001 standard deviation increase in the RWPP voting probability in the scenario of a high anti-globalisation attitude (Attitude = 10).

**Fig. 3:**
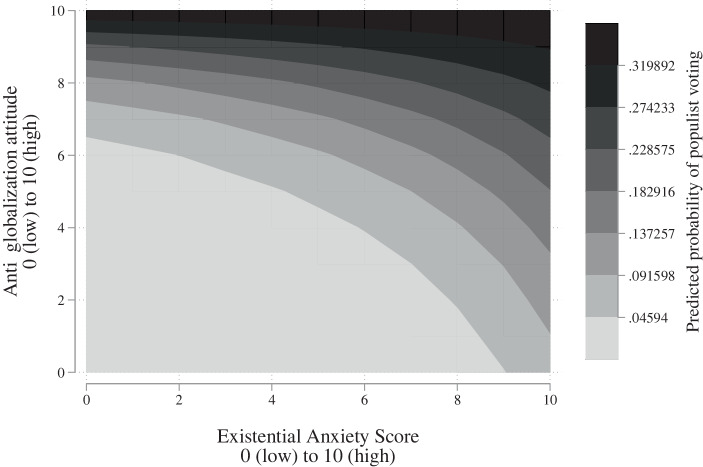
Predicted probabilities of RWPP voting calculated from interaction term

To analyse the effect of the interaction term further, Fig. 3 shows the predicted probability. The figure presents the effect of the interaction term along the values of EAS as well as those of the anti-globalisation attitude variable. In order to draw inference on the effect of the EAS in different scenarios of anti-globalisation attitudes, the figure can best be read by choosing a value on the y‑axis (anti-globalisation attitude) and moving to the right along the x‑axis (EAS). In general, a higher EAS is correlated with a higher RWPP voting probability across most of the values of the anti-globalisation attitude. As expected, higher EAS scores are however not correlated with a higher RWPP voting probability in a scenario of high anti-globalisation attitudes. This is exemplified by the fact that the predicted probability does not change across different EAS values, when the anti-globalisation attitude is above a value of 9. Similarly, the effect of the EAS is small in the case of low anti-globalisation attitudes, i.e., strong pro-globalisation attitudes.

The group of voters we are highly interested in are voters with moderate anti-globalisation attitudes, since we reason that those existential anxieties increases the likelihood of RWPP voting in this group of voters even though they do not hold strong anti-globalisation attitudes. In line with this, we see that the largest difference in the RWPP voting probability exists in the middle of the anti-globalisation dimension, with the predicted probability rising by 15 percentage points if the EAS changes from 1–10 at a constant moderate anti-globalisation attitude of 5. This finding supports hypothesis 2, demonstrating the mobilisation potential of RWPPs through fear-stoking among individuals with moderate globalisation predispositions.

In sum, the regression analyses and predicted probability plots provide enough support for our two hypotheses in statistical and substantial terms to conclude that (1) existential anxieties increase the probability of RWPP voting on the individual level and (2) the effect of existential anxieties on the RWPP voting probability is larger when the anti-globalisation attitudes are moderate.

## Robustness checks

In order to further assess our findings, we conducted several robustness checks for our regression analyses, which are displayed in Table 6 (see Appendix). First, we ran the regressions without robust standard errors and with various combinations of country and year fixed-effects. Second, we clustered the standard errors by country and year. Both variations did not affect the coefficients of our regression analyses in substantial terms. To account for the large sample size and its effect on the statistical significance of the coefficients we drew multiple random samples with 10% (about 8000–10,000 observations) of the observations in the full sample. The statistical significance nevertheless remained stable.

Besides using country fixed-effects we also account for the heterogeneity of our sample by conducting individual regressions for each year in our analysis. We calculated the statistical significance based on the model that only includes country and year fixed-effects (Model 1). The EAS and interaction term coefficient are stable across the different years. Based on the robustness checks, we conclude that our results are neither driven by certain specifications of the models nor by individual country-year-observations.

Due to the nature of our index and to avoid capturing other concepts with our choice of operationalisation, we ran additional regressions with different operationalisations. The results are displayed in Table 7 (see Appendix). The first model of the regression table shows the baseline model for reference. In second model the ‘traditions’ component is dropped from the exogenous dimension of our EAS with only a minor effect on the size of the coefficient. This finding supports the fact that the results for the EAS are not driven by the ‘traditions’ component. The third and fourth model show the results for the individual exogenous dimensions with the fourth model giving the coefficient for the exogenous dimension without the ‘traditions’ component. The coefficients again support the argument that the results are not driven by the ‘traditions’ and that the exogenous dimension in and by itself carries substantial weight.

For comparison, the fifth model shows the coefficient of the endogenous dimension, which is larger in size, underscoring the importance of political distrust and thereby populist attitudes in populist voting. The sixth model introduces both dimensions independently. Although the endogenous dimension carries some considerable weight in explaining populist voting, the coefficient of the exogenous dimension is nonetheless statistically significant. The combined coefficients of the exogenous and endogenous dimension are slightly smaller than the single coefficient of the EAS, supporting our argument that the need for security is dependent on the political distrust.

As for Eastern European countries, we calculated additional regression analyses of Model 1, i.e., without the control variable groups, for Bulgaria, Czech Republic, Estonia, Hungary, Slovenia, and Poland. While the EAS and the interaction term remain statistically significant in these countries, the substantial relevance of the EAS and the interaction term are considerably smaller than in the Western European sample countries.^12^ This supports our argument on systematic differences between Eastern and Western Europe and further justifies our chosen sample.

**Fig. 4:**
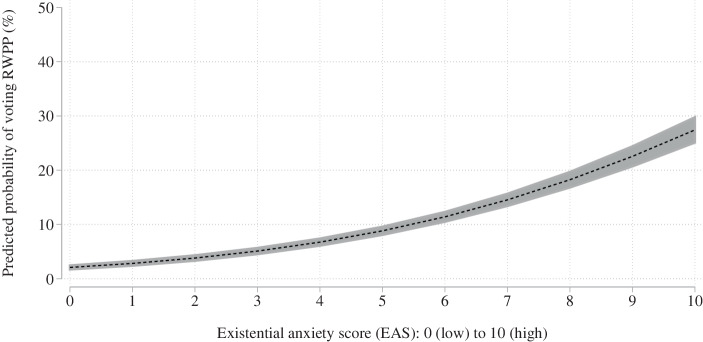
RWPP voting calculated from EAS with control variables

**Fig. 5:**
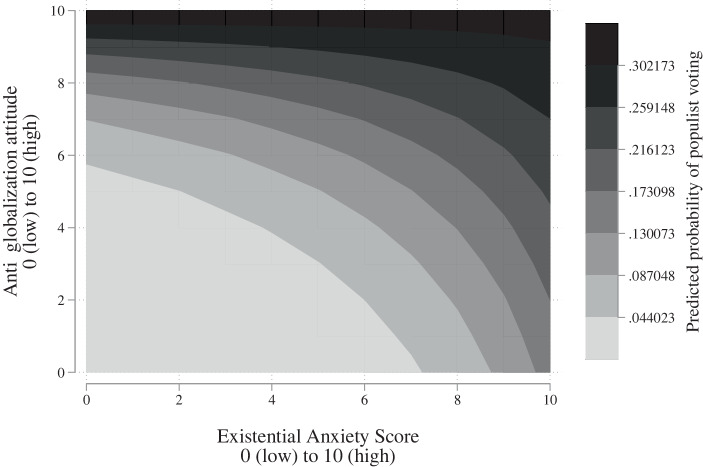
RWPP voting calculated from interaction term with control variables

We also again plot the marginal effects of the two regression analyses but with the full set of control variables. This does not change the predicted probabilities of RWPP voting across different EAS values although the confidence bands widen slightly due to the loss of observations (Fig. 4). The predicted probabilities of the models concerned with the interaction term is also not substantially affected by including the control variables, although the predicted probability slightly increases in the scenario of low anti-globalisation attitudes and high EAS values (Fig. 5).

In the last part of the robustness checks, we address the possibility of reverse causality. As indicated above, it is very likely that a reciprocal relationship exists between right-wing populist voting and experience existential anxieties. Since the ESS is not a panel dataset with the same individuals taking part in every round, we cannot test whether an individual first experiences existential anxieties and then votes for RWPPs or vice versa. From our theoretical considerations it nonetheless follows that the direction of causality at least also flows from experiencing existential anxieties to RWPP voting. To rule out the possibility that the direction of causality entirely runs from RWPP voting and the salience of RWPP issues in the political discourse, we regress the individual EAS on the lagged RWPP voting by country and year based on the previous ESS round. With this we can test whether the vote share/average support RWPPs experience at t − 1 is correlated with the individual EAS score of all voters as well as voter’s and non-voter’s of RWPPs. Running these regressions, we find no statistically significant correlation between country specific average RWPP support and individual existential anxieties. We conclude from this, that our results have to be viewed in the light of the reciprocal nature of the relationship between EAS and RWPP voting but that the results are neither from a theoretical nor an empirical perspective entirely driven by reverse causality.

## Conclusion

In this paper we addressed the research question as to why voters, why voters unconcerned by globalisation vote for anti-globalisation RWPPs. Our central argument was that existential anxieties make voters susceptible to crises narratives and securitisation agendas of RWPPs, particularly with regard to progressing globalisation, without being actually affected by globalisation nor having a profile of vulnerability.

To analyse the impact of existential anxiety on anti-globalisation RWPP voting, we constructed a novel existential anxiety index (EAS) based on ESS data. Using the EAS index, we assessed the empirical correlation between existential anxiety, anti-globalisation attitudes, and RWPP voting in 12 Western European countries between 2004 and 2008. We were able to show, first, that greater existential anxiety is linked to an increased likelihood of RWPP voting. Second, we demonstrated that existential anxiety especially mobilises voters with moderate globalisation attitudes to vote for anti-globalisation RWPPs.

One limitation of our analysis is the focus on Western European states and RWPPs. Our results can therefore not be transferred to Eastern Europe without any constraints. Further, we focused on right-wing populism but anti-globalisation attitudes and nationalist sentiments can also be found among left-wing populist parties (see Santana and Rama 2018; Zaslove 2008). Additionally, recent research by van Hauwaert and van Kessel (2018) as well as Hawkins et al. (2020) shows that populist attitudes are also an important predictor of populist voting, for which we were not able to control with the ESS data.

We also did not analyse the effect of EAS over time. In future research, it is crucial to use panel data to account for changes in existential anxieties and RWPP voting and to control for reversed causality. The latter is relevant as research has already shown that RWPP parties can affect political attitudes of voters (see Matthes and Schmuck 2017; Wirz et al. 2018), making it possible that RWPPs can also increase existential anxieties. At last, our index should be cross validated by using additional survey data.

Despite the remaining questions and discussed limitations, we provided first evidence why voters unconcerned by globalisation nonetheless vote for anti-globalisation RWPPs. We did so by moving beyond previous approaches and introducing individual sociopsychological factors into the equation. While voters seem to feel that globalisation disrupts the established traditional order of society and the role of the individual in it, the question remains whether RWPPs are even capable of providing the desired stability and how not overcoming traditional societal structures and cultural hegemonies can truly benefit any individual society or even voters of RWPPs.

## Appendix

**Table 4:** Description of variables. (Source: European Social Survey 2019; unless otherwise communicated)

**Main variables**	**ESS variables**	**Description**	**Meaning of variable’s highest value**	**Note**
*EAS*	Imptrad, impsafe, ipstrgv, trstplt, trstprl, trstprt	Existential Anxiety Score (EAS)	High existential anxiety	Aggregated
*RWPP vote*	Party voted for in the last election	RWPP voting (dummy)	Voted for an RWPP	Rooduijn et al
*Attitude*	Imueclt, euftf	Attitude towards globalisation	Very negative attitude towards globalisation	Aggregated
**Control variables**	**ESS variables**	**Description**	**Meaning of variable’s highest value**	**Note**
*Dem*	Gndr, agea, domicil, rlgdgr	Demographic profile	Female, age, living in the countryside, highly religious	–
*Socec*	Hinctnt, mbtru, eduyrs, unempl	Socioeconomic profile	In top 10% of income distribution, union member, years of education, unemployed	–
*Labr*	Offshore (isco88 + isco08), tradable (nacer 1 + nacer 11 + nacer 2)	Individuals with jobs that can easily be offshored or individuals in sectors which export or import goods (dummy)	Working in offshorable job, working in tradable sector	Coding based on Walter (2017); aggregated
*Status*	Statusseek (iprspot + ipshabt + ipsuces), hincfel, gincdif	Individual’s desire to be recognised by others	Highly status-seeking, income difficult, disagree strongly to gov. should reduce income differences	Based on Paskov et al. (2015); partly aggregated
*Polit*	Stfdem, lrscale	How satisfied with the way democracy works in country	Extremely satisfied with democracy, right on ‘left-right scale’	–

**Table 5:** Right-wing populist party coding

Country	Party	Party Name Full	ESS Rounds
Austria	FPÖ	Freiheitliche Partei Österreichs	2, 3, 7, 8, 9
Austria	BZÖ	Bundnis Zukunft Österreich	3, 7, 8
Belgium	VB	Vlaams Belang	2, 3, 4, 5, 6, 7, 8, 9
Belgium	FN	Front National	4, 5, 6, 7, 8
Belgium	Pp	Parti Populaire	5, 6, 7, 8, 9
Denmark	DF	Dansk Folkeparti	2, 3, 4, 5, 6, 7, 8, 9
Finland	PS	Perussuomalaiset	2, 3, 4, 5, 6, 7, 8, 9
France	RS	Resemblement National	2, 3, 4, 5, 6, 7, 8, 9
Germany	AfD	Alternative für Deutschland	7, 8, 9
Italy	LN	Lega Nord	6, 8, 9
Italy	FDI	Fratelli d’Italia	6, 8, 9
Netherlands	FvD	Forum voor Democratie	9
Netherlands	LPF	Lijst Pim Fortuyn	2, 3, 4
Netherlands	PVV	Partij voor de Vrijheid	4, 5, 6, 7, 8, 9
Norway	FrP	Fremskrittspartiet	2, 3, 4, 5, 6, 7, 8, 9
Sweden	SD	Sverigedemokraterna	5, 6, 7, 8, 9
Switzerland	LdT	Lega dei Ticinesi	2, 3, 4, 5, 6, 7, 8, 9
Switzerland	SVP	Schweizerische Volkspartei	2, 3, 4, 5, 6, 7, 8, 9
United Kingdom	UKIP	UK Independence Party	3, 7, 8, 9

The table shows the parties coded as far-right populist based on the PopuList of Rooduijn et al. (2019) and the anti-globalisation policy position measured by the CHES. An anti-globalisation attitude was coded if the respective party is positioned in the 25th percentile of the country specific position distribution with regard to EU integration and openness towards migration. The coding largely coincides with the far-right coding of Rooduijn et al. (2019) except for the Norwegian FrP and the Swiss LdT. The former has a comparatively moderate EU position in the CHES although being coded Eurosceptic in the PopuList and the latter not being included in the CHES but also coded Eurosceptic in the PopuList. We are aware of this discrepancy, but will keep both parties in the sample, as we follow the coding of the PopuList, which is designed more strongly for our purposes

**Table 6:** Robustness of results with different model specifications

Main variables	(1)	(2)	(3)	(4)	(5)	(6)
EAS	0.455*** (0.03)	0.459*** (0.03)	0.556*** (0.03)	0.459*** (0.08)	0.556*** (0.03)	0.717*** (0.10)
Attitude	0.784*** (0.03)	0.797*** (0.03)	0.779*** (0.03)	0.797*** (0.07)	0.779*** (0.03)	0.834*** (0.10)
EAS × Attitude	−0.059*** (0.00)	−0.061*** (0.00)	−0.055*** (0.00)	−0.061*** (0.01)	−0.055*** (0.00)	−0.070*** (0.02)
Country Fixed Effects	–	–	✓	–	–	✓
Year Fixed Effects	–	✓	–	–	–	✓
Country Clustered SE	–	–	–	✓	–	–
Year Clustered SE	–	–	–	–	✓	–
Random Sampled	–	–	–	–	–	✓
Observations	91,204	91,204	91,204	91,204	91,204	9096
Pseudo R^2^	0.107	0.116	0.187	0.116	0.187	0.194

Significance levels: *** *p* < 0.01, ** *p* < 0.05, * *p* < 0.10

Robust standard errors in parentheses

**Table 7:** Robustness of results with different EAS constructions

Main variables	(1)	(2)	(3)	(4)	(5)	(6)
EAS	0.452*** (0.01)	–	–	–	–	–
EAS Alt	–	0.418*** (0.01)	–	–	–	–
Exogenous Dim	–	–	0.137*** (0.01)	–	–	0.132*** (0.01)
Exogenous Dim. Alt	–	–	–	0.126*** (0.01)	–	–
Endogenous Dim	–	–	–	–	0.307*** (0.01)	0.304*** (0.01)
Country Fixed Effects	✓	✓	✓	✓	✓	✓
Year Fixed Effects	✓	✓	✓	✓	✓	✓
Observations	105,808	105,955	107,046	107,203	109,539	105,808
Pseudo R^2^	0.124	0.122	0.0898	0.0899	0.125	0.130

Significance levels: *** *p* < 0.01, ** *p* < 0.05, * *p* < 0.10

Robust standard errors in parentheses

## Footnotes

1 See summary statistics and Fig. 1.

2 While our argument relates to new RWPPs that focus on globalisation issues, this does not mean that classic far-right parties cannot also rely on psychological drivers for support or address certain components of ontological insecurity. See, e.g., Adorno et al. (1950) for one of the early receptions of the relevance of psychological factors.

3 The work of Oesterreich (e.g. 1997), who has psychometrically embedded authoritarianism and recorded it at the individual level, is also intriguing in this context.

4 Sample countries: Austria, Belgium, Denmark, Finland, France, Germany, Italy, the Netherlands, Norway, Sweden, Switzerland, and the United Kingdom.

5 Although the Spanish right-wing populist party Vox ran in elections prior to entering the parliament in 2019, the vote share of the party was so small that an indication of voting for Vox was categorised as “Other”.

6 To assess whether this exclusion is justified we run robustness checks with Eastern European countries after the analysis. Running the regressions for Bulgaria, Czech Republic, Estonia, Hungary, Slovenia, and Poland shows that our index remains a significant while the substantial relevance is reduced.

7 In general, the selection of variables in order to construct the index is not definitive. Thus, our specific compilation of variables is rather an assessment of the ESS items that capture both dimensions best. Alternatives items can or must be applied, when other surveys are used.

8 See Table 4 in the Appendix for a description of all variables.

9 According to the Kaiser-Meyer-Olkin (KMO) measure of sampling adequacy, which specifies a value above 0.5 to be acceptable (Kaiser 1974, p. 35), the present KMO-value above 0.7 justifies the performance of a PCA.

10 The additive approach is more straightforward and does not lead to different results as running an additional PCA for the two dimensions results in equal weights.

11 In the robustness checks we will also run a marginal effects plot with the inclusion of the control variables.

12 The detailed regression results for these additional robustness checks can be made available upon request.
